# Barriers to Mental Health Service Use among Palestinian-Arab Women in Israel: Psychological Distress as Moderator

**DOI:** 10.3390/ijerph191912557

**Published:** 2022-10-01

**Authors:** Fareeda Abo-Rass, Sarah Abu-Kaf, Ora Nakash

**Affiliations:** 1 School for Social Work, Smith College, Northampton, MA 01063, USA; 2 Conflict Management and Resolution Program, Ben-Gurion University of the Negev, Beer Sheva 84105, Israel

**Keywords:** psychological distress, mental health service use, women, minorities, Palestinians, Arabs, Israel, stigma-related barriers, attitudinal barriers, instrumental barriers

## Abstract

Background: Many studies indicate that ethnic minority women, including women from the disadvantaged Palestinian-Arab minority in Israel, experience higher rates of psychological distress but are less likely to use mental health services. This study examined psychological distress and its role as a moderator in the relationship between mental health service use and stigma-related, attitudinal, and instrumental barriers. Method: Cross-sectional study of 146 Palestinian-Arab women who completed measures of psychological distress, mental health service use, the Barriers to Care Evaluation scale, and sociodemographic characteristics. Results: Participants who did not utilize mental health services reported higher levels of all barrier types compared to participants who reported previous use, but lower levels of psychological distress. Psychological distress was a significant moderator only in the relationship between attitudinal barriers and mental health service use. Conclusions: This study highlights the role of psychological distress in the relationship between barriers to and utilization of mental health services, helping professionals and policymakers increase mental health service use among Palestinian-Arab women in Israel and other vulnerable women elsewhere.

## 1. Introduction

Psychological distress is defined as involving symptoms of anxiety and depression, social dysfunction, and inability to cope with daily activities [1]. Psychological distress and other mental disorders or symptoms are recognized as a global public health problem, impairing important areas of functioning and quality of life [2]. This recognition has intensified since the outbreak of the COVID-19 pandemic, as the rates of psychological distress and other mental health issues has increased significantly [3].

Numerous lines of evidence indicate that ethnic/racial minority women experience higher rates of mental health problems, including psychological distress, compared to women from ethnic majorities or even ethnic minority men [4,5,6]. For example, in 2014, the Cabinet Office Race Disparity Unit in the UK reported the findings of a survey, according to which nearly a third (29%) of Black minority women in the UK had experienced “a common mental health disorder” over the past working week; the respective ratios among White British or Other White women were 21% and 16% [7].

However, women from minority groups are less likely to use mental health services compared to majority women [8,9,10]. A report regarding mental health service use among different ethnic groups in the US from 2008–2012, by the Substance Abuse and Mental Health Services Administration, revealed that mental health service use was lower among Black and Hispanic women compared to White women, even among women with severe mental illness [11]. As shown below, this problem of greater distress and lower utilization of health services also occurs among women from the Palestinian-Arab (hereafter, Arab) minority, citizens of Israel. Surprisingly, though, it has attracted little scholarly attention, a gap addressed by the present study.

### 1.1. Arab Women in Israel: Demographic Background

Israel is officially defined as a “Jewish and democratic” state. According to the Israel Central Bureau of Statistics (CBS), most of its citizens are Jews, with a large, 21% Arab minority [12,13]. Members of the Arab minority are mostly Muslim, with significantly smaller Christian and Druze groups [14]. They are distinct from the Palestinians in the Occupied Territories in that they have Israeli citizenship. Most live in specific areas and within exclusively Arab towns and villages, with about 8% living in mixed Jewish-Arab cities [14].

In 2019, out of a total population of about 9 million citizens, 708,000 Palestinian Arab women aged 15 and over lived in Israel [12]. Among those aged 25 and above, the majority were married (peaking at 83.6% between the ages of 35 and 39) [15]. The fertility rate among Arab women continues to decline and today it is 2.82 births per woman, compared to 3 births per Jewish woman [16]. Nevertheless, Arab women still marry at a younger age and start a traditional family, with about half of all women being mothers of children under 17 (compared to 35% of Jewish women), and only about 7% heading single-parent families [17].

Compared to Jewish women, half of whom have some form of postsecondary education, only about a quarter (24.1%) of Arab women have higher education [17]. Nevertheless, in recent decades, there has been an increase in the percentage of Arab women with higher education. For example, the percentage of female Arab students in BA studies is almost 13% (compared to 45% of Jewish women), but they make up 66% of Arab undergraduate students in Israel (note, however, that this trend has still not affected postgraduate studies) [18].

### 1.2. Arab Women in Israel: Mental Health

The rates of mental health problems among the Arab minority in Israel are significantly higher than in the Jewish population [19,20,21,22]. In particular, Arab women suffer from greater psychological distress than do Jewish women. Kaplan et al. [10] found that Palestinian women in central areas in Israel have depression rates 2.5 times greater than do Jewish women. Two other Israeli studies found that Arab women not only suffered from psychological distress more than did Jewish women, but also more than did Arab men. The first study found that 45.7% of Arab women over the age of sixty reported high psychological distress, compared to 33% of Arab men and 25.2% of Jewish women [23]. The second study showed, based on the World Mental Health Survey, that the rates of any affective or anxiety disorder in the last 12 months were higher among Arab women (12%) than among Arab men (10.2%) or Jewish women (10.1%) [24]. Finally, a recent study based on extensive data collected by the Health and Environment Survey among Arab citizens of Israel shows that 41.1% of Arab women reported high psychological distress compared to 28.5% of Arab men [5]. Despite these alarming findings, to date the effect of psychological distress on the use of mental health services and its relationship with the barriers to such use in this population have not been investigated. This is despite the fact that previous studies have shown psychological distress to be significantly associated with mental health service use and with perceived barriers to service use among women and minorities [25,26,27].

More broadly, the political, economic, and sociocultural contexts of Arab society in Israel are important, interrelated determinants of psychological distress and mental health service use among women. The political context is the ongoing Palestinian-Israeli conflict, which involves both the Israeli military and the Jewish and Palestinian-Arab populations in Israel and the Occupied Territories. Combined with the experience of personal and structural discrimination, it means that Arab women are subjected to ongoing stress, affecting their physical and mental health beyond their individual problems [20]. This context also involves lack of government investments in infrastructure and education in Arab cities and towns [14].

In economic terms, compared to Israeli Jews, the Arab minority in Israel is disadvantaged in terms of most socioeconomic indicators: they have lower incomes, and higher levels of unemployment [28]. Additional problems narrowing the range of economic opportunities open to Arab women include lack of public transportation in Arab villages and cities, the need to master Hebrew in higher education and the labor market, the lack of job openings in Arab areas, and the enduring traditional structure of Arab society resulting in low rates of job market participation by women [20]. The employment rate of Arab women between the ages of 25 and 64 was 37.4% in 2019, and 30.1% for those aged 20–24—Significantly lower than among Jewish women of the same ages. These rates dropped further with the outbreak of the COVID-19 pandemic [29]. Arab women work mainly in education, health and welfare, where wage levels are relatively low. The wage level of Arab women is 32% lower than that of Arab men and 38% lower than that of Jewish women [29].

Regarding the sociocultural context, several factors affect the mental health and well-being of Palestinian women. In many respects, Arab society in Israel remains collectivist and traditional. Often, the demand to place the family and communities’ interests above individual interests is more pronounced for Arab women, who are considered solely responsible for the care of their nuclear families and even their parents and spouses’ parents [30]. This responsibility has diminished little with the increase in their rate of integration into the labor market or higher education [31]. Still, many Arab women in Israel are subjected to a double burden that affects their mental health. In addition to the demands of the modern job market, Arab society expects them to be disciplined, obedient daughters, good spouses, and devoted mothers. Additionally, in many cases, the family plays a big role in the timing of marriage and the choice of spouse [32], narrowing career options and potentially adding a major source of lifelong distress. Even under the best of circumstances, many Arab families still holds patriarchal values, adding yet another source of distress. Thus, Arab women in Israel may experience psychological distress not only because they are members of a disadvantaged ethnic minority but also because they are women in a traditional collectivist society [32].

### 1.3. Barriers to the Utilization of Mental Health Services among Arab Women in Israel

Despite the psychological distress of Arab women in Israel, the few available studies on this subject indicate that they underuse mental health services [24,33,34]. There appear to be three main reasons for that. The first reason is stigma: much like Arabs worldwide, members of the Arab minority in Israel are characterized by a tendency to stigmatize mental illness, the mentally ill, and mental health treatment [35,36]. In the Arab minority in Israel in particular, such attitudes have been found to be a barrier to formal mental health service use [37]. Regarding treatment, many Arabs ascribe mental difficulties to external or supernatural factors, as opposed to biological or psychological or biological factors [36,38]. Many perceive mental health issues as a divine “test” or “punishment” and seek religious in addition to or without seeking medical help [39,40]. Compared to men, Arab women are more likely to adhere to stigmatizing beliefs and attitudes concerning mental illness [33,41,42,43].

The second reason is that members of the Arab minority in Israel rely on informal help or prefer to cope on their own or within the family or community when it comes to mental health problems [37]. According to Ayalon et al. [33], to deal with mental health problems, Arab women rely on support from their nuclear and extended family, friends, or religious or self-help practices.

Finally, the third reason for the underutilization of mental health services among Arab women in Israel is instrumental and structural barriers. Despite being provided free of charge to all citizens under the universal healthcare law, mental health services in Israel are less available in Arab locales and are also of poorer quality. Combined with the shortage in public transportation services in these locales, accessing public or private mental health services in Jewish areas requires private vehicles and money [44]. Moreover, the existing mental health services are not culturally adapted—Even to the extent of being able to receive therapy in Arabic, given that they include relatively few Arabic-speaking professionals. The result is service underutilization as Arab patients tend not to seek treatment from Jewish therapists because of linguistic, cultural and political differences [20,35].

### 1.4. The Current Study

Arab women in Israel are a vulnerable group characterized by high levels of psychological distress and underutilization of mental health services. It is therefore crucial to help women from this minority to overcome barriers to seeking mental health services [33]. Accordingly, the first aim of the present study is to examine the level of psychological distress experienced by Arab women in Israel.

The second aim is to assess barriers to mental health service use—This is done using the Barriers to Access to Care Evaluation scale (BACE v3) [45]. BACE classifies potential barriers into stigma-related, attitudinal, and instrumental barriers. As we have seen, all play a significant role in the underutilization of mental health services by Arab women, making it an appropriate scale for examining the barriers thereto. This aim will be examined by comparing the barrier levels of women reporting using mental health services and those who do not.

The third aim of this study is to examine whether psychological distress moderates between the three types of barriers identified by Clement et al. [45] and the use of mental health services by Arab women in Israel. There is clear evidence that barriers to mental health service use affect service utilization. This relationship has been examined in the literature in the context of socio-demographic variables, but little is known about it in the context of clinical variables. We therefore explore the conditions of psychological distress under which this relationship becomes significant, especially since mental health in general has been found to moderate between different variables related to health outcomes and behaviors [46,47,48].

## 2. Materials and Methods

### 2.1. Participants and Procedure

We employed a cross-sectional research design. One hundred and forty-six women completed a self-report questionnaire. Beyond gender, the inclusion criteria were (a) being over the age of 18 and (b) being an Arab citizen of Israel. The study was approved by the Human Subjects Ethics Committee of Ben-Gurion University in Israel. Participants were approached using convenience sampling. All participants were from the northern and central regions of Israel, and thus did not include the important subgroup of Bedouin Arabs in southern Israel, since they have unique sociodemographic characteristics that affect mental health issues differently [20].

The first author initially recruited participants who had met the inclusion criteria and who varied in age, education, marital status, and area of residence, and then asked them to refer her to other potential participants (snowball technique). Prior to participating in the study, candidates sign an informed consent, then they were briefly asked about their sociodemographic characteristics to ensure that the first group of participants had diverse characteristics so that the snowball did not “roll down” to the same population category. Similarly, a questionnaire link includes on the first page an informed consent, was initially sent to several participants with diverse sociodemographic characteristics, who were asked to forward it to others. When a face-to-face meeting was held, the participants filled out the questionnaire independently; the first author intervened only to clarify questions as required, taking care to be objective and not to bias the participants’ answers.

This combined approach to data collection was adopted to ensure that not only internet users would fill out the questionnaire and thus perhaps bias the findings. We also wanted to include participants who did not feel comfortable participating in a mental health study in the presence of another person, given the mental health-related stigma prevalent in Arab society.

Both types of interviews used a structured survey form in Arabic that had been piloted with fifteen participants for clarity. The first page in both the online and hardcopy version included a description of the study, an informed consent form, and the researchers’ contact details. Only participants who had signed the form or checked a box to indicate their agreement to participate could then complete the full survey. Because of the study’s online component, we could not determine the response rate. *t*- and χ^2^ tests were conducted to examine differences in the main study’s variable between participants from the two recruitment strategies, and no significant difference was found (*p* > 0.05).

### 2.2. Measures

#### 2.2.1. Barriers to Access to Care Evaluation (BACE v3)

The 30-item BACE v3 [45] was used to assess barriers to mental health service use. Each of the items refers to a specific barrier known to have delayed or prevented an individual from seeking professional mental healthcare. The scale includes three types of barriers: (1) Stigma-related (12 items; for example, “Concern that I might be seen as crazy”); (2) Attitudinal (10; for example, “Wanting to solve the problem on my own”); and (3) Instrumental (8; for example, “Problems with transport or travelling to appointments”). All items are rated on a 4-point Likert scale (from 0 = not at all to 3 = a lot); six included the additional option “not applicable”. The total scale score has high validity, reliability, and acceptability [45].

Following previous practice [26,49], three subindices were generated for each scale type by averaging the relevant items, with higher scores indicating a more significant barrier (0–1 indicated a weak barrier; 1–2 a medium barrier; and 2–3 a severe barrier). The scale was translated into Arabic using the back-and-forth method [47,48]. The translated version showed high internal consistency for all barrier types (Cronbach’s α = 0.88 for stigma-related, 0.75 for attitudinal, and 0.86 for instrumental barriers).

#### 2.2.2. Psychological Distress—General Health Questionnaire (GHQ-12)

Psychological distress was assessed using the General Health Questionnaire (GHQ-12) [50], which contains 12 items rated on a 4-point Likert scale. The scores of seven items were reversed. Sample items were “Have you recently lost much sleep over worry?” and “Have you recently been thinking of yourself as a worthless person?” Following the original scoring method, an overall index was calculated by summing the scores of all items, such that the scoring method becomes 0, 0, 1, and 1 instead of 1, 2, 3, and 4, respectively, providing scores ranging from 0 to 12, with higher scores representing higher levels of psychological distress. The scale is available and validated in Arabic [51]. The internal reliability of the scale in the current study was high (α = 0.78).

#### 2.2.3. Demographics

The participants’ sociodemographic characteristics were examined using a questionnaire that collected details about their age, years of education, marital status (single, married, divorced, widowed), Hebrew proficiency (poor, average, excellent), religiosity (not religious, somewhat religious, religious, devout), and income (above average, average, or below average in Israel). Respondents also reported whether they had used mental healthcare services, by responding with Yes or No to the question: Have you sought help from formal mental health services (such as a psychologist’s or psychiatrist’s clinic, a mental health center or clinic affiliated with your HMO, a mental health clinic in a general or mental health hospital, a psychotherapist’s or a social worker’s clinic, or formal online mental help services)?

### 2.3. Data Analysis

The collected data were analyzed using SPSS-25 and PROCESS macro [52]. Two sets of analyses were conducted. First, descriptive statistics were used to characterize the participants’ and main research variables. To assess differences in sociodemographic characteristics, psychological distress, and barriers to mental health service use between participants using and not using mental health services, *t*- and χ^2^ tests were conducted.

Second, moderator effects of psychological distress were estimated with the PROCESS macro for SPSS, controlling for the effects of education, as it was found significantly associated with mental health service use (the dependent variable). All continuous variables were standardized, using the centered scores of the variables divided by the standard deviations.

## 3. Results

### 3.1. Participant Characteristics

Table 1 presents sociodemographic characteristics of participants by mental health service utilization history. Most participants were married. Their mean age was 35.82 years (*SD* = 10.02), and their mean years of education were 14.32 (*SD* = 4.97). Fifty-five (37.67%) of the participants reported previous mental health service use. Significant differences between mental health service users and non-users were found only in education, with participants with more years of formal education reporting higher rate of previous use of mental health services.

### 3.2. Differences between Service Users and Non-Users in Psychological Distress and Barriers to Service Use

To meet the first and second aims of the study regarding level of psychological distress and barriers to mental health service use, Table 2 lists the range, means and standard deviations of psychological distress, stigma-related, attitudinal, and instrumental barriers, as well as differences between participants who reported using vs. not using mental health services. As can be observed, statistically significant differences between the two groups were found in all three barrier types. Participants who did not utilize mental health services in the past reported higher levels of all barrier types compared to participants who reported previous use of mental health services. In addition, there was a significant difference in psychological distress levels between women who used mental health services and those who did not, with the former reporting moderate levels of psychological distress whereas the latter reporting lower levels.

### 3.3. Psychological Distress as a Mediator between Use of Mental Health Services and the Barriers Types

To meet the third aim of the study and assess the moderating effect of psychological distress on the relationship between barriers and service utilization, we performed three sets of moderation analyses in PROCESS. The first model includes stigma-related barriers as an independent variable; the dependent variable was mental health service use; and the moderating variable was psychological distress. The second moderation model included attitudinal barriers as an independent variable; the dependent variable was mental health service use, and the moderating variable was psychological distress. Finally, the third model included instrumental barriers as an independent variable; the dependent variable was mental health service use, and the moderating variable was psychological distress.

Only the second moderation model has been found significant, which means psychological distress was a significant moderator only in the relationship between attitudinal barriers and mental health service use. Table 3 shows this moderation effect. Our results indicate a significant negative association between attitudinal barriers and mental health service use; this association is weaker when psychological distress is high (*B*(*SD*) = −2.2929 (0.6146), *p* = 0.000, 95% CI = −3.49–−1.08) than when it is low (*B*(*SD*) = −4.7843 (1.38), *p* = 0.000, 95% CI = −7.49–−2.07).

## 4. Discussion

The main aim of this study was to examine the psychological distress levels of Palestinian-Arab women in Israel and the stigma-related, attitudinal, and instrumental barriers to their mental health service use. Specifically, we explored the role of psychological distress in the relationship between these barriers and the use of mental health services. The findings showed that psychological distress levels were low to moderate among both mental health service users and non-users. The findings showed that psychological distress levels were low to moderate among both mental health service users and non-users. Considering the existing literature about the disadvantaged socioeconomic and political context of Palestinian-Arab women in Israel, and a recent study showing high levels of psychological distress among them [5], we expected to find higher levels of psychological distress in our sample. This finding could be explained in terms of the education level of the women in our sample, relatively high compared to the population of Arab women in Israel [17], especially since previous studies showed a negative relationship between psychological distress and education [53,54,55], including specifically among Arab women in Israel [5].

A possible explanation for the difference between the findings of our present study and a previous study that found high psychological distress is that our sample did not include Bedouin women from southern Israel, whereas Khatib’s study [5] included women from all regions, showing that Bedouin women reported higher psychological distress, affecting the average distress among the entire sample. Other studies pointed to a relatively high frequency of mental health issues among Bedouin women [20], who face additional economic, political, and social challenges. For example, about one-third of the Bedouin women still live in so-called “unrecognized villages” that lack the most basic infrastructure, including in some cases running water and electricity [56].

Regarding the role of psychological distress, it has been found to be a significant moderator in the relationship between attitudinal barriers and mental health service use. This means that the relationship between attitudes to mental health and the use of mental health services is stronger when the psychological distress is lower. Whereas when the distress is high, the association between this barrier and mental health service use is significantly weaker. However, it was not found to be a moderating factor between stigma-related or instrumental barriers and mental health service use. This finding attests to the power of these barriers in the lives of Arab women in Israel, so that regardless of distress levels, there is a relationship between stigma-related and instrumental barriers and mental health service use, such that the women’s psychological distress may not propel them to overcome barriers to seek help. This finding makes sense in groups such as the Arab minority in Israel, with its low socioeconomic status, low availability of mental health services, and highly stigmatizing approach to mental health treatment [35,36]. The findings are aligned with the experience of Arab women, who experience additional disadvantages compared to men [29], making it particularly difficult for them to overcome these barriers.

The role of psychological distress in the relationship between the three barrier types and the use of mental health services raises an interesting issue: when the barrier is more external (i.e., stigma of Arab society and culture, or instrumental barriers), even high psychological distress may not moderate it. Conversely, when the barriers are internal or personal, distress levels have some effect on the relationship between attitudinal barriers mental health service use. This insight highlights the urgent need for institutions to take responsibility for addressing external barriers, and dismantling structural obstacles to accessing mental health care for the Arab minority in Israel. Among other things, they can lead campaigns to raise mental health awareness and remove the social stigma that constitutes a significant barrier for that minority. Institutions also need to improve Arab women’s attitudes to mental health service utilization by improving mental health literacy [57,58,59], which was found to be significantly related to mental health service use in this population [37].

Another point that emerges from the finding regarding the role of psychological distress in the relationship between attitudinal barriers and mental health service use is that women who seek mental healthcare probably suffer from severe distress. Ideally, women should seek psychiatric treatment before their condition deteriorates, given the association between the severity of psychological problems at treatment entry and treatment outcomes [60]. Accordingly, improving the attitudes of Arab women to mental healthcare also has preventive value.

Lastly, our findings show that both mental health service users and non-users reported relatively low levels of stigma-related, attitudinal, and instrumental barriers. This was also unexpected since, in general, studies indicate high barriers to mental health service use among ethnic minorities [45,61,62,63]. Previous studies have also shown, however, that gender and education (women and better-educated) are significantly related to the use of mental health services [37,64], which may explain the findings of the current study. We also think that the sociocultural changes taking place in Arab society in Israel improve mental health literacy, and attitudes to mental illness, its treatment, and stigma [35], factors that may also explain the low perceived barriers among Arab women in our sample. Finally, the low levels of psychological distress among the participants may also explain this finding: Bustamante et al. [26], for example, found that mental health symptoms and perceived need for mental health treatment are positively related with perceived barriers to mental health service utilization.

### 4.1. Limitations and Future Directions

This study has several limitations. First, its use of a convenience and culturally homogeneous sample limits the generalizability of our findings and conclusions. As it includes only Arab women from central and northern Israel, its findings may not be applicable to the Bedouin Arab women in the south. Future studies should also include Bedouin Arab women and examine whether the role of emotional distress differs across the different subgroups of Arab society in Israel. Second, our cross-sectional design prevents us from inferring causal relationships. Third, our data are based mostly on self-report measures administered online, which are limited in preselecting high internet users and the potential for inaccurate interpretation of questions. Conversely, the portion of our data collected in face-to-face interviews might be affected by social desirability bias. Finally, the BACE scale does not examine barriers that may be unique to the specific sociopolitical context of Arab women in Israel, such as religiosity, familialism, mental health literacy, micro-aggression or political barriers Future studies must consider further barriers to better understand mental health service use in this unique group.

### 4.2. Theoretical and Practical Implications

Theoretically, our findings expand on the existing knowledge regarding psychological distress and barriers to mental health service use among women from minority groups. They also add to the literature regarding factors that moderate the relationship between the barriers and the use of mental health services. Thus, our findings can help understand other factors that affect the relationship between the barriers and mental health service use among women from disadvantaged ethnic minorities. Our study also contributes to the literature by linking barriers directly to actual service use, as opposed to help-seeking intentions or behaviors.

Practically, this study informs professionals and policymakers about the barriers to mental health service use among Palestinian-Arab women in Israel and about their psychological distress, drawing their attention to the fact that distress does not moderate the relationship between stigma-related or instrumental barriers and mental health service use, so that the relationship between these two barrier types and the use of services remains significant regardless of distress. This sensitizes policymakers to the crucial role of these two barrier types among Arab women and calls upon them to develop intervention programs to remove these two barriers in order to increase service use. However, psychological distress has been found to moderate the association between attitudinal barriers and mental health service use, drawing professionals’ attention to the unique clinical characteristics of Arab women or women from minorities with similar characteristics, who are able to overcome their attitudes and reach out to treatment.

## 5. Conclusions

The importance of the current study is twofold: in examining the role of psychological distress in the association between barriers and mental health service use, and in focusing on Palestinian-Arab women in Israel—a vulnerable group with unique characteristics. The findings show that psychological distress among women from a minority group can moderate between attitudinal barriers and mental health service use, but not between instrumental and stigma-related barriers and mental health service use. This indicates the possible mediating role of psychological distress between barriers and healthy behaviors such as seeking mental health services and shows for the first time that clinical characteristics—and not only sociodemographic or socio-psychological factors—can moderate such a relationship. Finally, it highlights the significance of both external and internal barriers and the clinical characteristics of those who overcome attitudinal barriers in particular.

## Figures and Tables

**Table 1:** Participants’ Sociodemographic Characteristics.

	Entire Sample (*n* = 146)	Reported Using Mental Health Services (*n* = 55)	Reported Not Using Mental Health Services (*n* = 91)	
Marital status, n (%)				
Single	30 (20.5)	9 (16.3)	21 (23)	
Married	106 (72.6)	41 (74.5)	65 (71.5)	NS
Divorced	9 (6.2)	4 (7.3)	5 (5.5)	
Widowed	1 (0.7)	1 (1.9)	0 (0)	
Income, n (%)				
Above average	37 (25.3)	15 (27.3)	22 (24.2)	
Average	61 (41.8)	26 (47.3)	35 (38.5)	NS
Below average	48 (32.9)	14 (25.4)	34 (37.3)	
Hebrew proficiency, n (%)				
Poor	5 (3.4)	3 (5.4)	2 (2.1)	
Average	65 (44.5)	26 (47.3)	39 (42.9)	NS
Excellent	76 (52.1)	26 (47.3)	50 (55)	
Religiosity, n (%)				
Not religious	18 (12.3)	5	13	
Somewhat religious	45 (30.8)	16	29	NS
Religious	75 (51.4)	30	45	
Devout	8 (5.5)	4	4	
Education years, *M* (*SD*)	14.32 (4.97)	15.62 (3.91)	13.54 (5.38)	*t*_(138.93)_ = −2.69 **
Age, *M* (*SD*)	35.82 (10.02)	36.61 (9.87)	35.34 (10.13)	NS

Note. ** *p* < 0.01.

**Table 2:** Differences between Service Users and Non-Users in Stigma-Related, Attitudinal, and Instrumental Barriers.

	Range	Reported Using Mental Health Services (*n* = 55)	Reported Not Using Mental Health Services (*n* = 91)	
Psychological distress, *M* (*SD*)	0–12	5.27 (2.13)	2.41 (2.19)	*t*_(144)_ = −7.69 ***
Stigma-related barriers, *M* (*SD*)	0–3	0.04 (0.10)	0.26 (0.41)	*t*_(96.18)_ = 4.82 ***
Attitudinal barriers, *M* (*SD*)	0–3	0.08 (0.17)	0.27 (0.03)	*t*_(134.43)_ = 6.09 ***
Instrumental barriers, *M* (*SD*)	0–3	0.08 (0.15)	0.27 (0.45)	*t*_(109.83)_ = 3.56 ***

*Note.* *** *p* < 0.001.

**Table 3:** Moderation Effect of Psychological Distress in the Relationship between Barriers to Mental Health Services and Service Utilization.

	*B*(*SE*)	*p*	95% CI
Education years	0.94 (0.32)	0.000	−3.37–−0.93
Psychological distress	2.53 (0.48)	0.000	1.57–3.47
Attitudinal barriers	−3.54 (0.03)	0.000	−5.43–−1.66
Psychological distress X Attitudinal barriers	1.28 (0.49)	0.009	0.31–2.24
Cox R^2^	0.65		

## Data Availability

The data presented in this study are available on request from the corresponding author.
